# Characteristics and clinical significance of CD163+/CD206+M2 mono-macrophage in the bladder cancer microenvironment

**DOI:** 10.3906/biy-2104-17

**Published:** 2021-10-18

**Authors:** Xiangjie KONG, Ming ZHU, Zhirong WANG, Zhuoqun XU, Jianfeng SHAO

**Affiliations:** Department of Urology, Wuxi People’s Hospital Affiliated to Nanjing Medical University, Wuxi China

**Keywords:** Bladder cancer, tumor microenvironment, mono-macrophage, CD40

## Abstract

The tumor microenvironment may recruit monocytes, with a protumoral macrophage phenotype (M2) that plays an important role in solid tumor progression and metastasis. Therefore, it is necessary to understand the characteristics of these cells for cancer prevention and treatment. Bladder cancer tissue samples and paracarcinoma tissues samples were collected, and the expression of CD163^+^ cells in tumor tissues was observed. Then, we observed the expression of infiltrating CD45^+^CD14^+^CD163^+^ cell subset and analyzed the molecular expressions related to immunity and angiogenesis. C57/BL6 mice were inoculated subcutaneously, and dynamic changes of CD11b^+^F4/80^+^CD206^+^ mononuclear macrophages expression for tumor-bearing mice were detected. The results showed that the proportion of CD45^+^CD14^+^CD163^+^ mono-macrophage subset infiltrated by tumor tissue was significantly higher than that in paracarcinoma tissues. In bladder cancer tissue, the expression rate of CD40 in CD45^+^CD14^+^CD163^-^ mono-macrophage subset was significantly lower than that in CD45^+^CD14^+^CD163^+^ mono-macrophage subset. Similar results were found in the paracarcinoma tissues. We found that, as the proportion of CD11b^+^F4/80^+^CD206^+^ mono-macrophages increased gradually, the difference was statistically significant. CD163^+^/CD206^+ ^mono-macrophages in bladder cancer microenvironment are abnormally elevated, and these cells are closely related to tumor progression. CD40 may be an important molecule that exerts biological function in this subset.

## 1. Introduction

Bladder carcinoma is one of the most common urologic malignancies worldwide and is a heterogeneous disease . In 2019, the American Cancer Society estimated 80,470 new cases of bladder cancer and 17,670 deaths due to bladder cancer in the United States . In China, the incidence and mortality rates of bladder cancer have gradually increased in recent years . Noninvasive, well-differentiated tumors have a relatively indolent natural history, whereas poorly differentiated tumors are prone to invade and metastasize. The clinical importance of metastasis, the mechanisms facilitating the progression from benign to invasive and finally to metastatic cancer remain poorly understood. Bladder cancer and other solid tumors contain a number of infiltrating immune cells, predominantly macrophages (Maniecki et al., 2012).

High density of M2 mono-macrophages was significantly associated with late clinical staging in patients with bladder cancer. In response to different environment stimuli, macrophages can appear as a range of different phenotypes . The expression of CD163 mono-macrophages in bladder cancer tissue are believed to play a key role in the growth, progression and metastasis of tumors by producing growth factors, proteases and cytokines, which promote neoangiogenesis, connective tissue breakdown, scavenging of cellular debris and tumor-cell proliferation . Compared with CD68, CD163 is regarded as a highly specific monocyte/macrophage marker for M2 macrophages . Mono-macrophages infiltrating the microenvironment of tumors are usually labeled with the leukocyte marker CD45, and the mono-macrophage marker molecules CD14 and CD45^+^CD4^+^ flow gate strategy defines tissue infiltrating monocyte macrophages (Vidal et al., 2012; Sylvester et al., 2018). Here, CD45 was used to detect leukocytes, rather than examine CD14, as CD45 can eliminate the interference of other cells to the greatest extent, and the measurement is more accurate (Coppin et al., 2017; Sylvester et al., 2018). CD40 is an important costimulatory molecule that plays an important role in T cell immune regulation. CD40 is mainly expressed on the surface of B lymphocytes, mono-macrophages and dendritic cells. CD40L is mainly expressed on the surface of T cells and is a CD40 ligand. On the one hand, CD40-CD40L, which plays an important role in antiviral and anticancer responses, regulates the T cell immune response. On the other hand, CD40 can activate mono-macrophages and release inflammatory mediators. CD40-CD40L also plays a key role in mediating angiogenesis and tumor growth (Gutierrez et al., 2019). CD206, a carbohydrate-binding receptor expressed by select populations of macrophages, dendritic cells and nonvascular endothelium, is a heavily glycosylated molecule, and its N-glycosylation sites are highly conserved between humans and mice. CD206 function can be altered through proteolytic cleavage and changes in glycosylation and conformation , which is also a typical marker of M2 macrophages. In recent years, extensive research has suggested that angiogenesis in bladder cancer contributed to tumor growth and progression . By collecting bladder cancer tissue samples, the expressions of CD163^+^ cells and related membrane molecules in the mono-macrophage subset were analyzed, and the relevant mechanism and clinical significance of how this mono-macrophage subset exerted biological function were discussed. 

## 2. Materials and methods

### 2.1. Materials

The anti-human antibodies were purchased from US Biolegend and US R&D System and US Biolegend. Cell Lines: Mouse-derived long-term cell strain MB49 cells were derived from American Merck and placed in a 5% CO_2_ humidified incubator for subculturing (MB49 cells were derived from C57BL/Icrf-a’ mouse bladder epithelial cells that were transformed by a single 24-h treatment with DMBA). 

### 2.2. Flow cytometry program for bladder cancer tissue of patients

(1) Eight patients with primary bladder cancer were included in this study. Biopsies were obtained from transurethral tumor resections, and aliquots were frozen immediately. Samples for histological examination were removed before freezing. Two patients received radical cystectomies, and 6 patients were treated by transurethral tumor resections and received intravesical pirarubicin therapy. The patient clinical data are summarized in Table. Tissue cell processing: the cancerous tissue and distal non-cancerous tissue of the same bladder cancer patient was collected (confirmed by pathology as normal tissue, at least 2cm away from the edge of the cancer). The study was reviewed and approved by the Ethics Committee of the First Affiliated Hospital of Soochow University, and all patients signed the informed consent (2016 Ethics (application) Lot No. 000-1); the tissues were washed with precooled PBS to remove free erythrocytes. After being cut into pieces, they were placed in 1640 culture medium (volume 1 mL) containing 0.1% collagenase IV (Sigma, USA) and incubated in a 37 °C incubator for shaking reaction for 1 h. Subsequently, the resuspended pre-chilled phosphate buffered solution (PBS) (5 mL) was ground, and then cell resuspension solution was obtained; after that, 30μm filter (Miltenyi Biotec, Germany) was used to remove islet-like cell cluster to obtain a single cell suspension. (2) Expression of CD163^+^ mono-macrophage in the tissue: make gates by anti-CD45^-^PC7^+^anti-CD14^-^FITC antibody, mono-macrophages infiltrated by the tissue and defined as CD45^+^CD14^+^ were measured by flow cytometry. Experimental group: anti-CD45^-^PC7^+^anti-CD14^-^FITC was as basic anti-body; anti-CD163^-^APC was added to the experimental group; anti-IgG-APC was added into the control group; after being mixed, make reactions on ice for 30 min, being washed with PBS for once; after being resuspended, CD45^+^CD14^+^ was the characteristic of mono-macrophage expression to make gates, and the expression of infiltrating CD45^+^CD14^+^CD163^+^ mono-macrophage subset in bladder cancer and adjacent tissues was detected. (3) Comparative analysis of CD163^+^ and CD163^-^ mono- macrophage phenotype: on the basis of the research approach above, CD163 antibody was used to make gates, the expression of membrane molecule Tie-2, PD-L1, CD31 and CD40 as well as VEGFR2 and other molecules in CD45^+^CD14^+^CD163^-^ and CD45^+^CD14^+^CD163^- ^mono-macrophage subset were analyzed by flow cytometry, and meanwhile immunoglobulin G (IgG) was set as the same type of control. Specific streaming operation scheme was the same as the former.

**Table:** Clinical data and patient characteristics.

Variables	No. of patients(bladder cancer)	%	No. of patients*(paracarcinoma)	%
Patients	8		3	
Age (years)				
Median	66.13		72.67	
Range	50-81		65-81	
Gender				
Male	6	75.0	2	66.7
Female	2	25.0	1	33.3
Clinical stage of tumor				
Ta	5	62.5	2	66.7
T1	2	25.0	1	33.3
T2-T4	1	12.5	0	0
Tumor grade				
I/II	5	62.5	2	66.7
III/IV	3	37.5	1	33.3
Tumor size				
< 3 cm	6	75.0	3	100
≥ 3 cm	2	25.0	0	

Abbreviations: Ta, superficial tumors; T1, connective tissue-invasive tumors; T2-T4, muscle-invasive tumors ﹡ Stage and grade in the paracarcinoma group were determined by bladder cancer.

### 2.3. Fluorescence microscopy 

(1) Slice preparation: fresh bladder cancer tissues were dehydrated respectively with 10%, 20% and 30% (v/v) in gradient sucrose for 24 h after being fixed with 4% paraformaldehyde overnight; dehydrated tissue in optimal cutting temperature (OCT) compound was placed in –80 °C low temperature refrigerator for preservation. The frozen tissue was cut into continuous slices with 7-μm thick by a Leica Freezing Automatic Microtome and placed in a –20 °C low-temperature refrigerator for storage. (2) Antibody staining: lyophilized sections were soaked in ice-cold acetone for 10 min, then let it dry at room temperature, and drew the outline of the tissue with a PAP pen; Sections and 5% bovine serum albumin (BSA) were sealed for 1 h at room temperature; the section area was completely covered with fluorescent antibody (CD14^-^FITC and CD163^-^APC) after being diluted by 10-fold 0.1% BSA (about 100μL), and the reaction was shaken for 1 h at 4 °C. After being soaked thoroughly in PBS for 3 times, it (the section) was sealed with 4’, 6-diamidino-2-phenylindole (DAPI) glycerol solution. (3) Microscopy: the expression of CD163 was observed by Leica fluorescence microscope (× 400), and records and pictures were made.

### 2.4. Construct bladder cancer tumor-bearing mouse model

A total number of 18 adult female C57BL/6 mice with weights in the range of 16–20g (Shanghai Slack Laboratory Animal Co., Ltd.) were raised in the specific pathogen free (SPF) experimental animal center and were provided by the Laboratory Animal Center of Soochow University with the license number SYXK(Su) 2012-0045 (2016 Ethics (application) Lot No.000-2). The murine bladder cancer cell line MB49 was expanded in vitro, and the cell density was adjusted to 1×10^7^/ml, which was implanted subcutaneously in the groin of C57BL/6 mice (200 μL per mouse). After the tumors were formed, a group of experimental mice were euthanized and observed every three days. All mice were anesthetized with isoflurane (1.5%) inhalation and sacrificed via cervical dislocation. Euthanasia can be confirmed with cardiac arrest and mydriasis in experimental animals. Animals will be euthanized when the maximum diameter of the tumor will not be above 20 mm. And the tumor tissues were peeled off. The average time of mouse tumor model was 19 days. The duration of the whole experiment was half a year. After being photographed, the tissue cells were treated. 

### 2.5. Dynamically detect the expression of CD206+ macrophages of tumor-bearing mice in vivo

After the tumor tissues was peeled off, washed once with PBS, and cut to small pieces 1 mm in diameter, the tissue fragments were resuspended in 20% bovine serum 1640 culture medium and treated with a gentle MACS tissue processor (Miltenyi Biotec, Germany). Then the tissue fragments were removed by a 150 mesh filter, centrifuged and resuspended (1500 rpm in centrifugal speed, 16.2 cm in centrifugal radius). Thereafter, it was removed by a 30μm filter (Miltenyi Biotec, Germany) to remove islet-like cell cluster to obtain single cell suspension. Multi-color flow cytometry scheme was established with CD11b^-^FITC, F4/80^-^PC7 and CD206^-^PE single standard tubes. On this basis, CD11b^-^FITC^+^F4/80^-^PC7^+^IgG-PE was taken as negative control tubes and CD11b^-^FITC^+^F4/80^-^PC7^+^CD206^-^PE as test tubes. The cell suspension was mixed with the antibodies above, and then it was placed on ice for 30 min. After that, the reaction was stopped by being washed with PBS (2mL) once. It was resuspended in PBS solution (0.5mL) again. CD11b^+^F4/80^+^ was taken as the characteristics of making gates, and the dynamic expression of type M2 mononuclear macrophages characterized by CD206 in cancer tissues of tumor-bearing mice was analyzed by flow cytometry. 

### 2.6. Statistical methods

Statistical analysis and drawing were performed using GraphPad Prism 5.0 (version 5.01). Intergroup analysis was paired Student’s t-test or One-way ANOVA; phenotypic analysis of CD163^- ^and CD163^+^ mononuclear macrophages was ANOVA; Bonferroni was used following the ANOVA; p < 0.05 showed the difference was statistically significant.

## 3. Results

### 3.1. Expression ratio of CD163+ mono-macrophage in bladder cancer tissue

**Figure 1:**
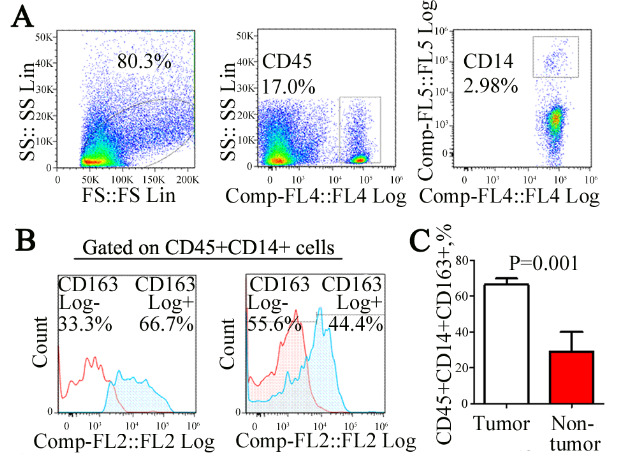
Expression ratio of CD163+ mono-macrophage in bladder cancer tissue. Mono-macrophages infiltrating the tissue were detected by flow cytometry (A). Notably, 80.3% of living cell enrichment regions in total single cell suspension were obtained from tumor tissue homogenate. Approximately, 17.5% mononuclear macrophages were detected in 80.3% of leukocytes in the preceding gate. In total, 2.98% mononuclear macrophages were detected in 17.5% of leukocytes in the preceding gate. The expression of the CD45+CD14+CD163+ mono-macrophage subset in bladder cancer tissues (n = 8) was significantly higher than that in adjacent normal tissues (B and C) (n = 3, 67.88 ± 7.2% vs. 27.05 ± 11.18%, p = 0.001).

Mono-macrophages infiltrated in tissue were detected by flow cytometry (Figure 1A). The expression proportion of CD45+CD14+CD163+ mono-macrophage subset in bladder cancer tissue (n = 8) was significantly higher than that in adjacent normal tissues (n = 3, 67.88 ± 7.2% vs. 27.05 ± 11.18%, P = 0.001) (Figures 1B and 1C). It is prompted that CD45+CD14+CD163+ mono-macrophage subset is significantly increased in bladder cancer microenvironment, which is one of the characteristics of tumor immune microenvironment. 

### 3.2. Phenotypic identification of CD163- and CD163+ mono-macrophage subset in bladder cancer tissue

**Figure 2:**
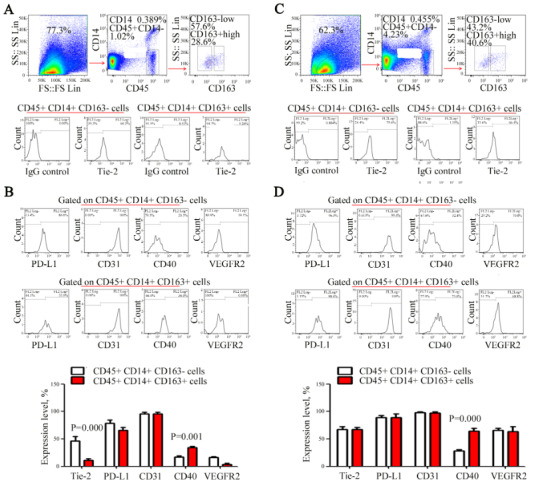
Phenotype identification of CD163- and CD163+ mono-macrophage subsets in 8 bladder cancer tissues (A) and 3 adjacent tissues of 3 bladder cancer cases (C). Then, the expression levels of Tie-2, CD31, pd-l1, CD40 and VEGFR2 in CD163- and CD163+ mono-macrophage subsets were detected (%) and statistically analyzed (B and D). There was no significant difference between the two groups in CD31, PD-L1 and VEGFR2 and other molecules (p = 0.686, 0.613, 0.452, respectively) (A and B). There was also no significant difference between the two groups in Tie-2, CD31, PD-L1, VEGFR2 and other molecules (p = 0.885, 0.836, 0.808, 0.713, respectively) (C and D).

Monocyte macrophages are mainly involved in immune regulation and neovascularization in the tumor microenvironment (Arnold et al., 2019). Tie-2, CD31 and VEGFR2 are associated with neovascularization (Loges et al., 2007; Xu et al., 2019). PD-L1 and CD40 are important immunoregulatory molecules that are closely related to the immune response of tumors. Therefore, we chose the above molecules (Goto et al., 2019). The expression rate of Tie-2 in CD45^+^CD14^+^CD163^-^ mono-macrophage subset (43.8 ± 10.12%) was significantly higher than that in CD45^+^CD14^+^CD163^+^ subset (12.71 ± 3.06 %) (p = 0.000) (Figure 2A). However, the expression rate of CD40 in CD45^+^CD14^+^CD163^-^ mono-macrophage subset (19.1 ± 2.51%) was significantly lower than that in CD45^+^CD14^+^CD163^+^ subset (33.91 ± 2.8%) (p = 0.001) (Figure 2B). There was no significant difference between the two groups in CD31, PD-L1 and VEGFR2 and other molecules (p = 0.686, 0.613, 0.452) (Figure 2B). This suggested that CD163^+^ mono-macrophage subset was manifested as the phenotype of Tie-2^low^CD40^high^ in cancer tissues.

### 3.3. Phenotype identification of CD163- and CD163+ mononuclear macrophage subset in adjacent tissues of bladder cancer

In contrast to the cancerous tissue, the expression rate of CD40 in CD45^+^CD14^+^CD163^-^ mono-macrophage subset (24.71 ± 2.54%) was significantly lower than that in CD45^+^CD14^+^CD163^+^ mono-macrophage subset (67.9 ± 5.66%) (p = 0.000). There was no significant difference between the two groups in Tie-2, CD31, PD-L1, VEGFR2 and other molecules (p = 0.885, 0.836, 0.808, 0.713) (Figure 2C and 2D). This suggested that the CD163^+^ mono-macrophage subset in normal adjacent tissues was only manifested as the phenotype of CD40^high^. For the comparison of cancer tissues and adjacent tissues, CD40^high^ is a common feature of CD163^+ ^mono-macrophage subset, suggesting that this molecule may play an important role in biological functions of the CD163^+^ mono-macrophage subset. 

### 3.4. Dynamic expression of CD206+ mononuclear macrophage in bladder cancer tumor-bearing mouse model

**Figure 3:**
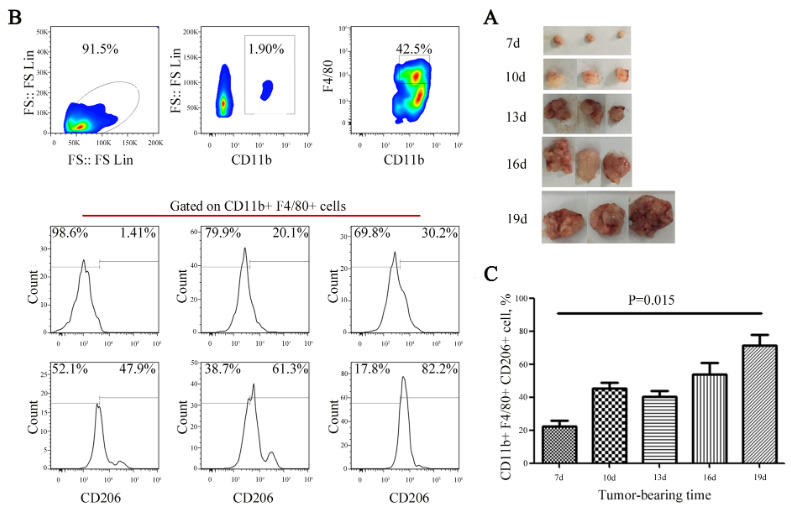
Dynamic expression of CD206+ mono-macrophages in a bladder cancer tumor-bearing mouse model. (A) With the growth of the tumors, three mice were euthanized in batches by cutting off necks on the 7th, 10th, 13th, 16th and 19th days after tumor implantation. The tumors are shown at different points in time. (B and C) Mono-macrophages were delineated by CD11b+F4/80+, and the expression changes in the CD206+ subset were detected and statistically analyzed. The results showed that the proportion of infiltrating CD206+CD11b+F4/80+ mono-macrophages gradually increased with tumor growth (p = 0.015).

Construct tumor-bearing mouse model using MB49. With the growth of the tumors, three model mice were euthanized in batches by cutting off necks on the 7th, 10th, 13th, 16th and the 19th day after tumor implantation. The tumor tissues were peeled off, and the tumor sizes were recorded (Figure 3A); maximum diameter of tumor was 18 mm, each mouse had only one tumor. After tumor tissues were treated as single cell suspension, they were counted by flow cytometry. Mono-macrophages were delineated by CD11b^+^F4/80^+^, and the expression changes of CD206^+^ subset were detected (Figure 3B). The results showed that the proportion of CD206^+^CD11b^+^F4/80^+^ infiltrated by mononuclear macrophage subset gradually increased with tumor growth (p = 0.015) (Figure 3C), but there was a downtrend of the proportion of CD206^+^CD11b^+^F4/80^+^ cells on Day 13 that we will find out the reason by expanding the sample size.

## 4. Discussion

Tumor microenvironment is the “soil” on which tumor cells depend, and provides support for the growth and spread of cancer cells (“seeds”) . The components that make up the tumor microenvironment are relatively complex, including fibroblasts, stromal stem cells, endothelial cells, and immune cells. The highly concerned immunological factors are the myeloid derived cells represented by mono-macrophages . Mono-macrophages in the tumor microenvironment can promote tumor growth and metastasis in the following several ways . (1) In the aspect of tumor immune escape, as important antigen-presenting cells, mono-macrophages at the tumor infiltration site can express a large number of negative costimulatory molecules, exhibit the characteristics of low expression of human leukocyte antigen DR (HLA-DR), which plays an important role in inhibiting antigen-specific T cells response. (2) In the aspect of angiogenesis, mono-macrophages promote the angiogenesis of tumor vessels and lymphatics by expressing vascular (lymphatic vessel) growth factors such as vascular endothelial growth factor (VEGF). In addition, they can directly participate in the differentiation of tumor vessel endothelium as precursor cells to promote tumor angiogenesis. (3) In terms of invasion and metastasis, mono-macrophage plays an important role in invasion and metastasis of tumor by secreting metalloproteinases, reducing the adhesion between tumor and ground substance and promoting the degradation of basement membrane. (4) In terms of tumor formation “pre-metastatic niche”, tumor metastasis is not random, and only a few specific organs may form metastases. Like all solid tumors, bladder cancer requires an active angiogenesis to support its growth and progression, Bladder tumor-associated angiogenesis is emerging as an important prognostic factor and represents a promising potential therapeutic target for cancer treatment. Mono-macrophages in the tumor microenvironment can reach the pre-metastatic organs prior to the tumor, and create an immune microenvironment suitable for tumor growth. This tumor pre-metastatic microenvironment composed of mono-macrophages and other myeloid derived cells is called “Pre-metastatic Niche” . Thus, it can be seen that mono-macrophages in the tumor microenvironment play a major role in promoting tumor invasion and metastasis.

Monon-macrophages are highly heterogeneous groups whose phenotypes may vary in different microenvironment. This study found that the expression of CD40 in CD45^+^CD14^+^CD163^+^ mono-macrophage subset was significantly higher than that in CD45^+^CD14^+^CD163^-^ mono-macrophage subset in both cancer tissues and adjacent tissues (Figures 2B and 2D), suggesting that CD40 may play an important role in the biological function for this subset. Enormous research suggests that the signal mediated by CD40-CD40L can induce APC activation. CD40-CD40L signal can also upregulate the expression of inflammatory cytokines such as Interleukelin-6 (IL-6), VEGF and tumor necrosis factor-α (TNF-α), participate in tumor angiogenesis and promote tumor invasion and metastasis . Recent studies show that the expression level of CD40 is closely related to its biological effects . CD40 with a low expression level can promote cell proliferation and be exempted from the apoptosis; CD40 with a high expression level can induce apoptosis. Additionally, it has been reported that CD40 plays a key role in myeloid-derived suppressor T cell (Treg) expansion (Pan et al., 2010). 

Angiopoietin-1 (Ang-1) and angiopoietin-2 (Ang-2) can interact with Tie-2, and Ang-1/Tie-2 signaling promotes the formation of vascular endothelium and lumen. Ang-2 can compete with Ang-1 for binding to Tie-2, thereby compete to inhibit angiogenesis. Tie-2 mainly promotes angiogenesis; mono-macrophages characterized as Tie-2^+^ are also presumed to be involved in the neovascularization of tumors. In the present study, the expression rate of Tie-2 in the CD45^+^CD14^+^CD163^-^ mono-macrophage subset was significantly higher than that in the CD45^+^CD14^+^CD163^+^ subset (Figures 2A and 2B). These results suggested that M1-like mono-macrophages may play a more important role in promoting angiogenesis than do M-2-like mono-macrophages.

It was well known that TAMs with an M2-like phenotype (markers CD163, CD204, and CD206) promote tumor growth while M1-like TAMs (CD68, CD80, and CD86) may inhibit tumor growth . In this study, we found that the number of CD206+ mononuclear macrophage subset gets bigger along with the tumor growth (Figures 3B and 3C). Here, we speculate that CD206 may become a therapeutic target in bladder cancer, and clinically we can also actively detect the expression of CD206 to calculate tumor load, determine treatment responses, and observe patient prognosis (Wang et al., 2020).

In summary, we compared and analyzed the expression characteristics of mono-macrophages in the bladder cancer microenvironment. Functional experiments require the adequate tumor infiltration of mono-macrophages in bladder cancer, which restricts functional research on bladder cancer. We will strive to improve this part of our follow-up work. Our results showed that CD40 is highly expressed in M2-like mono-macrophages and that Tie-2 is highly expressed in M1-like mono-macrophages. The phenotype of M2-like mono-macrophages was identified as CD45^+^CD14^+^CD163^+^CD40^high^Tie-2^low^ for the first time, which established a foundation for further studies on the biological function of this subgroup. As well, CD206 might be an important therapeutic target that needs to be validated in more bladder cancer samples.
